# Where Next?

**Published:** 1897-09

**Authors:** 


### WHERE NEXT?

THE thirty-fourth annual meeting of the United States Veterinary Medical Association will largely influence the place of meeting for 1898. The Executive Committee will have to decide whether Nashville can be considered an Eastern, Western, Central, or distinctly a Southern meeting. We had supposed that as it was so far removed from what was for so many years its chief Eastern centre that it would naturally fall to the East in 1898. We had learned of Boston being in the field for next year's meeting, but she will first have to win a decision that Nashville is a Western meeting. Nashville presents a new phase in Association affairs, and as it is distinctly a Southern meeting there must necessarily be a Northern section of our land yet untouched, and we are glad to note a new face in the field, no less a worker and hustler than our esteemed colleague, Dr. M. E. Knowles, of Montana; and he means to have heard at Nashville the claims of this wonderfully beautiful and important section of our country. Not to be lagging in the race, Colorado and Indiana are joining hands to take the meeting West next year. Surely no better signs of a healthy, progressive growth of our Association need be presented than this, and the friendly competition for place is a happy sign of the times. *There is room for all newcomers in the field.*

